# Editorial: DNA-based population screening for precision public health

**DOI:** 10.3389/fgene.2022.1061329

**Published:** 2022-10-24

**Authors:** Laura V. Milko, Muin J. Khoury

**Affiliations:** ^1^ Department of Genetics, University of North Carolina at Chapel Hill, Chapel Hill, NC, United States; ^2^ Office of Genomics and Precision Public Health, Centers for Disease Control and Prevention, Atlanta, GA, United States

**Keywords:** DNA, population screening, precision public health, health equity, implementation science, genome sequencing, expanded newborn screening

## Introduction

Rapid advances, increasing availability, decreasing costs of sequencing technologies, computational pipelines for variant interpretation, and training of clinical personnel, are accelerating the integration of genomic sequencing into routine health care.

Although genomic sequencing has demonstrated utility as an indication-based diagnostic tool for certain diseases, the full potential of DNA sequencing as a non-diagnostic tool for population-level screening is not yet realized. DNA-based population screening has enormous potential to identify people with underlying genetic predisposition to serious diseases such as cancer and heart disease, who represent 1–2% of the population (Murray et al., 2020). Early detection, disease prevention, and timely treatment can improve health outcomes and equity, and usher in a new era of precision public health (Khoury et al., 2018a).

Nevertheless, the ascertainment of otherwise apparently healthy individuals with underlying genetic risk will necessitate a departure from the traditional model of familial or personal risk-based genetic testing in specialty settings to a population-based model of screening in primary care or community settings (Bean et al., 2021). Additionally, adoption of a population-level genomic screening strategy requires dismantling barriers to equitably enact such an approach in the context of clinical care, design and conduct, to develop a sufficient evidence base for clinical utility and cost-effectiveness (Roberts et al., 2019).

Given the low frequency of individuals with a heritable genetic risk, sharing of study methods and data from evidence-gathering pilot studies are needed to foster collaborative linkage of observations and outcomes to address these gaps (Khoury et al., 2018b). With the ever-increasing number of settings carrying out DNA-based screening, this Research Topic of the journal commissioned articles to highlight the breadth of perspectives and approaches that comprise the current state of knowledge about DNA-based population screening, including genome sequencing data and interpretation, data governance and stewardship issues, stakeholder engagement, patient and provider education, and clinical outcomes from ongoing clinical and research programs in a variety of settings.

## Utilizing implementation science frameworks

DNA-based population screening is increasingly viewed through the lens of implementation science methods and frameworks (Bangash and Kullo, 2020). Use of rigorous methods to mitigate barriers to equitable uptake, evaluation of the impact on providers and health systems, and aggregation and sharing of patient health outcome data are increasingly relied upon to support the translation of effective DNA-based screening practices into routine clinical care to improve public health.

In this Research Topic of the journal, (Wildin et al.) describes feasibility testing of the Genomic Population Health Pilot Program within the University of Vermont Health Network using the well-known Consolidated Framework for Implementation Research (CFIR). The article details the barriers to and facilitators of this unique program that was among the first non-research DNA-based screening pilots. (Jones et al.) detail the use of the RE-AIM implementation science framework (Reach, Effectiveness, Adoption, Implementation, and Maintenance) to conduct separate pragmatic program evaluations of two different Geisinger DNA screening pilots, the MyCode community health research program and a primary care clinical DNA screening pilot, based on their most relevant and informative domains.

The systematic review by (Shen et al.) of multi-level barriers, facilitators, stakeholder perceptions, and outcomes of implementing DNA-based population screening supports the need for more research to address significant barriers to health equity, ethical, legal, and social implications (ELSI), readiness for implementation in primary care, and evidence gaps regarding clinical utility and long-term outcomes. Emphasis on the development of metrics for the collection and sharing of aggregated patient, health service, and intervention outcomes is critically important for evaluating the public health impact and cost-effectiveness of DNA-based population screening.

## Maximizing clinical utility

Currently available evidence does not provide support for the widespread use of predictive genomic screening in healthy populations. Thus, an inherent challenge for DNA-based population screening programs is determining which disease-causing genes and genomic variants to screen for to maximize clinical utility and minimize undue harms to healthy individuals. Incomplete penetrance and variable expressivity of genetic variants can result in a broad spectrum of phenotypes, from subclinical manifestations to severe disease, even among relatives harboring the same disease-causing genotypes. Our current understanding of the natural history of many genetic diseases is based on small cohorts of clinically diagnosed individuals, which raises valid concerns about overdiagnosis and overtreatment in unselected populations. (Kingdom and Wright) address this urgent need for a broader genotype-based understanding of risk identification with a comprehensive review of emerging clinical studies of common and rare genetic variation and its effect on human diseases.

Longitudinal data from clinical cases with positive results are also needed to reclassify potential pathogenic variants and link successful standards of clinical care to ascertainment by population-scale implementation of DNA-based screening. The work of (Wilhelm et al.) illustrates the value of combining longitudinal health information from follow-up genetic testing of screen-positive newborns with accompanying clinical information to inform genotype-phenotype correlations and reevaluate the clinical relevance of genetic variant data. (Ashenhurst et al.) highlight the predictive utility and complementarity of polygenic scores combined with other types of screening data such as family health histories, for providing an earlier and more precise diagnosis in high-risk individuals.

Cascade screening in blood relatives for a variant that confers an inherited disease predisposition is an important and cost-effective strategy for identifying and improving health outcomes of other at-risk individuals; however, there are substantial barriers to widespread acceptance of this beneficial process. In their manuscript (Schmidlen et al.) describe the impact of a proband indication on the uptake of cascade testing by family members based on two settings, one in which the proband has a clinical condition and presents for testing in a diagnostic setting as well as a non-diagnostic scenario where the proband was detected *via* proactive screening. (Haas et al.) evaluate whether an alternative approach to population genomic screening—automated sharing of family health history *via* the electronic health record (EHR)—offers an efficient and cost-saving method to facilitate cascade testing.

## Understanding public perceptions and values

Understanding the factors that affect public interest in participating in genomic research will ultimately support informed decision-making and minimize enrollment barriers in clinical offerings. (Roberts et al.) observe an association between awareness of genetic testing and educational attainment level and public interest in participating in genomic screening to learn about inherited predisposition to cancer. (Kaphingst et al.) investigate about whether offering genomic screening as part of routine health visits would stimulate interest and participation by ethnically diverse young women. (Brown et al.) explore the perceptions of parents who belong to underrepresented groups in genomic research in making an urgent and difficult choice about whether to enroll in the prenatal arm of the California-based Program in Prenatal and Pediatric Genome Sequencing (P3EGS), part of the Clinical Sequencing Evidence-Generating Research (CSER) consortium. Building on this work, (Outram et al.) reports on the expectations of the parents who ultimately did decide to enroll in the P3EGS study and the subsequent value to them of the prenatal genomic sequencing results they received.

## Prioritizing health equity in population screening

As (Azriel et al.) note in their article, the implementation of any health care innovation is generally accompanied by concerns about adequate reach and representation of medically underserved individuals. DNA-based population screening is subject to these concerns due to stark inequities posed by numerous barriers at the patient, provider, and policy levels. However, if the implementation of DNA-based population screening can be effectively moored to public health screening frameworks and community partnerships that center equity and justice as Azriel et al. describe, there is tremendous potential to improve outcomes for all individuals with inherited predispositions to certain actionable medical conditions, add to our knowledge base about the natural history and spectrum of disease in underrepresented populations, and potentially reduce the access gap to clinical and genetic services. In the article by (Powell et al.), a collaborative team of parents and researchers illustrate the development of a bidirectional partnership in which community stakeholders are integrated in the design, implementation, and dissemination of knowledge throughout the lifespan of the Age-Based Genomic Screening (ABGS) project. Engagement marketing concepts can foster these types of trust-based relationships with communities that have been historically marginalized in biomedical research to ensure that health disparities are not perpetuated in DNA-based population screening programs, as (Lewis et al.) describe from their engagement experiences with the *All of Us* program. (Rahimzadeh V. et al.) share a protocol for understanding public beliefs and values about stewardship of cloud-based human genomic data that can help to assuage concerns about data access and privacy.

## Expanding newborn screening to include genomic screening

Newborn screening (NBS) is a highly successful public health screening program for which early detection and effective interventions have resulted in established health benefits over many decades. Implementing DNA-based screening could significantly expand the number of conditions that NBS could screen for, and the gap between enhanced diagnostic capability and available, effective treatments is rapidly closing. However, effective and equitable implementation of expanded NBS incurs an even higher burden of evidence than screening healthy adults. (Armstrong et al.) examines the perspectives of parents of healthy newborns in the BabySeq Project who were surveyed about various aspects of newborn genome sequencing, including whether it should be state-mandated and accompanied by informed consent, and the return of different types of genetic information. (Brower et al., 2022) reports findings from the NBS Expansion Study and (Chan et al.) highlights opportunities for modeling to address the challenges of accelerating the process of adjudicating candidate conditions. (Pichini et al.) describe the development of an ethics- and engagement-informed Genomics England-sponsored Newborn Genomes Program to explore the utility of offering whole genome sequencing (WGS) in the newborn period.

## Addressing informed consent, education, and ELSI for expanded genomic NBS

Despite the expected benefit of rapidly emerging new therapies and the critical importance of early initiation of treatment for maximizing health benefits, widespread clinical integration of expanded genomic NBS has been effectively stalled due to substantial ethical, social, and practical challenges inherent in sequencing newborns. Historically, NBS has employed an “opt-out” model of consent due to its vast public health importance; however, expanding NBS by hundreds of conditions will concomitantly expand the range, relevance, and recommendations for the results parents might receive and will likely require parents to “opt-in” to expanded genomic NBS. This paradigm shift will entail educating parents on a broad array of relatively complex topics in preparation for informed decision-making and consent. Health care practitioners will require education and innovative resources for facilitating informed decision-making, parental consent and return of results. (Peay et al.) describe the development and evaluation of an electronic and patient-centered education and informed consent approach for the large-scale expanded NBS Early Check study. (Rahimzadeh V. et al.) balance the potential benefits against the possible harms in their assessment of unresolved challenges associated with using universal sequencing as a methodology for population screening of newborns. (Spencer and Fullerton) explore the ethical rationale for coinciding age of screening implementation for highly actionable genetic conditions with the age of maximum clinical utility in the general population.

## Building effective governance and infrastructure

DNA-based population screening has the potential to transform the practice of health care from reactively treating disease symptoms to proactively identifying at-risk individuals in the population and delivering precision care to prevent the onset of disease. Encapsulated in this Research Topic are articles describing broad advancement in research and clinical integration of DNA-based population screening. Creating and utilizing effective infrastructure to translate research to clinical practice remains crucial to realizing actual improvements in public health. The EHR features prominently in patient-centered healthcare as an important data tool for sharing results between providers and patients, monitoring clinical follow up, and, more recently, providing passive and active clinical decision support. (Elhanan et al.) describe barriers to relevant clinical action following the delivery by the Healthy Nevada Program of important genetic findings directly into participants’ EHR and proposes potential solutions centered on providing additional education and support for healthcare providers.

Advances in EHR functionality notwithstanding, the necessary infrastructure to enable learning healthcare systems remains elusive. Fertile settings for discussion and problem solving are needed to harmonize collection, analysis, and reporting of data and outcomes. The National Human Genome Research Institute’s Genomic Medicine XIV virtual meeting entitled; “Genomic Learning Healthcare Systems” provides promising support for priority research areas. (Roberts et al.) highlight outcomes from *The Transdisciplinary Conference for Future Leaders in Precision Public Health,* a participatory forum to accelerate solutions for precision public health challenges. Finally, (Onstwedder et al.) summarize necessary translational improvements required in practice and policymaking to operationalize the promise for DNA-based population screening for precision public health.

In conclusion, while currently available evidence does not provide support for the widespread use of predictive genomic screening in healthy populations the scientific, ethical and implementation foundation for such an endeavor is slowly being built. However, there is a significant need for more research to address significant barriers to health equity, ethical, legal, and social implications (ELSI), readiness for implementation in primary care, and evidence gaps regarding clinical utility and long-term outcomes. This research should use an implementation science framework and build effective governance and infrastructure. We hope our readers find the collection of papers herein useful in advancing the dialogue on DNA-based population screening towards a new era of precision public health.
